# Study on GIS-based suitability evaluation of the landscape environment of celebrity former residences in Huanggang

**DOI:** 10.1038/s41598-026-51878-0

**Published:** 2026-05-08

**Authors:** Haonan Sun, Yong Hua

**Affiliations:** School of Art and Design, Hubei University, Wuhan, 430062 China

**Keywords:** GIS, Celebrity former residences, Landscape environment, AHP, Suitability evaluation, Ecology, Ecology, Environmental sciences, Environmental social sciences

## Abstract

Celebrity former residences, as cultural carriers shaped by historical evolution, embody profound cultural significance and hold immeasurable historical, social, political, and economic value. Grounded in the theories of human settlement science, settlement geography, and landscape ecology, this study takes the preserved celebrity former residences in Huanggang City as its research objects. Using the Analytic Hierarchy Process (AHP) and adopting a geographical perspective, eight geographical evaluation factors were selected in ArcGIS to represent topography, hydrology, ecology, and geomorphological stability as assessment indicators. By analyzing the relationship between these geographical factors and the spatial distribution of the residences, a suitability evaluation system for the landscape environment of celebrity former residences in Huanggang was established. The results indicate that, among the 136 officially protected celebrity residences in the city, 66 are located in highly suitable areas, accounting for 48.53%; 49 are located in suitable areas, accounting for 36.03%; 18 are located in less suitable areas, accounting for 13.24%; and 3 are located in unsuitable areas, accounting for 2.20%. In addition, a case study of the former residence of Li Siguang in Tuanfeng County demonstrates that its siting highly corresponds to the established evaluation framework. These findings suggest that geographical environmental factors are not only closely linked to the site selection of celebrity residences in Huanggang but also provide valuable references and guidance for future architectural siting and landscape environmental planning.

## Introduction

Celebrity former residences commemorate historical figures who exerted significant influence in fields such as politics, culture, science, and the arts. These sites possess a unique cultural spatial character, with physical carriers including residences, courtyards, and studies, which are often preserved in their original state or restored to reflect a specific historical period. As tangible witnesses of historical events and important vehicles for cultural transmission and collective memory, they constitute a vital part of urban cultural heritage^1^. Existing research on celebrity residences has primarily concentrated on their cultural value, emphasizing their historical significance and the necessity of preservation. From a typological perspective, they can be categorized into seven groups, including national leaders, patriots, political figures, historical figures, cultural celebrities, scientists, and artists, with the majority belonging to political, historical, and cultural categories. This distribution highlights the central role of historical, cultural, and artistic dimensions in shaping urban memory. Current scholarship largely focuses on the cultural and historical value of celebrity residences, underlining both the urgency of their protection and their role as witnesses of urban history^2–4^.

The GIS (Geographic Information System) provides a powerful technical framework for spatial data management, spatial analysis, and visualization, offering strong support for evaluating the siting and landscape environment of celebrity residences^5^. Compared with traditional qualitative descriptions, GIS enables the integration of multi-source data and quantitative analysis, leading to more scientific and intuitive research outcomes.

Existing studies applying GIS-based suitability evaluation methods can be classified into several domains. Research on waste site selection and solid waste disposal emphasizes environmental safety and resource utilization^6–8^. Agricultural and soil suitability studies construct multidimensional systems of soil, climate, and crops to evaluate production potential and ecological carrying capacity, thereby optimizing agricultural layouts^9–12^. Ecological environment and restoration studies integrate landscape ecology and geostatistical theories to identify restoration suitability and risk patterns of ecosystems such as green spaces^13–15^. Urban space and land use evaluations focus on human settlements and public spaces, establishing integrated frameworks that combine natural, cultural, and social factors to reveal spatial suitability and its coupling with demographic and economic structures^16–23^. Research on ecological and cultural tourism integrates natural attractiveness, cultural value, and accessibility to assess spatial differentiation and propose differentiated development strategies^24–26^. Disaster risk and special scenario studies address issues such as refugee camp siting, natural hazards, and marine spatial planning, often combining multi-criteria decision analysis with machine learning to propose safe siting and ecological red line delineations^27–29^. Heritage and cultural landscape conservation, through the integration of GIS and multi-source datasets, has advanced the understanding of ancient settlement evolution^30^, proposed methods for prioritizing the conservation and restoration of historic buildings^31^, facilitated real-time monitoring of heritage environments^32^, and promoted the systematic management and digital transformation of diffuse cultural landscapes^33^. These studies primarily rely on GIS and multi-source datasets to reveal the evolutionary patterns of human–environment interactions. They propose methods for establishing conservation priority frameworks for architectural heritage, and construct integrated systems encompassing monitoring, management, and decision-making. Furthermore, they explore approaches for the database-driven and network-based management of cultural landscapes.

AHP, as a multi-criteria decision-making tool, provides a scientific basis for the comprehensive evaluation of complex spatial systems^34^. The suitability evaluation of settlement environments not only helps identify limiting factors in production and living spaces, thereby supporting land consolidation and spatial planning decisions^35^, but also offers an important theoretical approach for exploring human residential choice mechanisms and the evolution of human–land relationships^36^. Therefore, applying environmental suitability evaluation methods to the siting of historical celebrity residences can help interpret the relationship between traditional site-selection wisdom and the natural geographical environment, and provide scientific references for the conservation and spatial planning of traditional settlements. At the same time, the study analyzes the suitability of natural environmental conditions for human settlement at the regional scale. It is not merely intended to determine where celebrity residences should have been located, but to examine whether their existing spatial distribution corresponds to areas of high suitability, thereby revealing the environmental logic of traditional site selection. Compared with existing studies that focus on functional site selection or development suitability, this study regards the spatial distribution of historical celebrity residences as spatial samples of optimally selected human settlement environments in historical periods. From the perspective of cultural heritage, it examines the issue of environmental suitability for human habitation and attempts to establish a link between the two, thereby revealing the environmental logic of traditional settlement site selection at the regional scale and providing a new quantitative analytical approach for cultural heritage spatial studies.

Building on the perspective of historical heritage and cultural landscape protection, this study conducts a suitability evaluation of the landscape environment of celebrity former residences in Huanggang City, treating the spatial distribution of preserved celebrity former residences as representative samples of historically preferred human settlement environments. The research follows the theories of human settlement science and settlement geography, employing GIS spatial analysis combined with the AHP. Eight geographical evaluation factors were selected to represent topographic features, ecological environment, hydrological conditions, and geomorphological stability. These indicators were used to construct a suitability evaluation system for the landscape environment of celebrity residences in Huanggan, and to analyze the relationship between their spatial distribution and the patterns of environmental suitability. Through weighted factor analysis, spatial overlay, and suitability zoning, the study quantitatively examines the spatial distribution patterns of celebrity residences^37^, and explores the relationship between the spatial distribution of celebrity former residences and the regional environmental suitability patterns. Finally, the evaluation framework is empirically validated through a case study of the former residence of Li Siguang.

## Methodology

### Study area

**Fig. 1:**
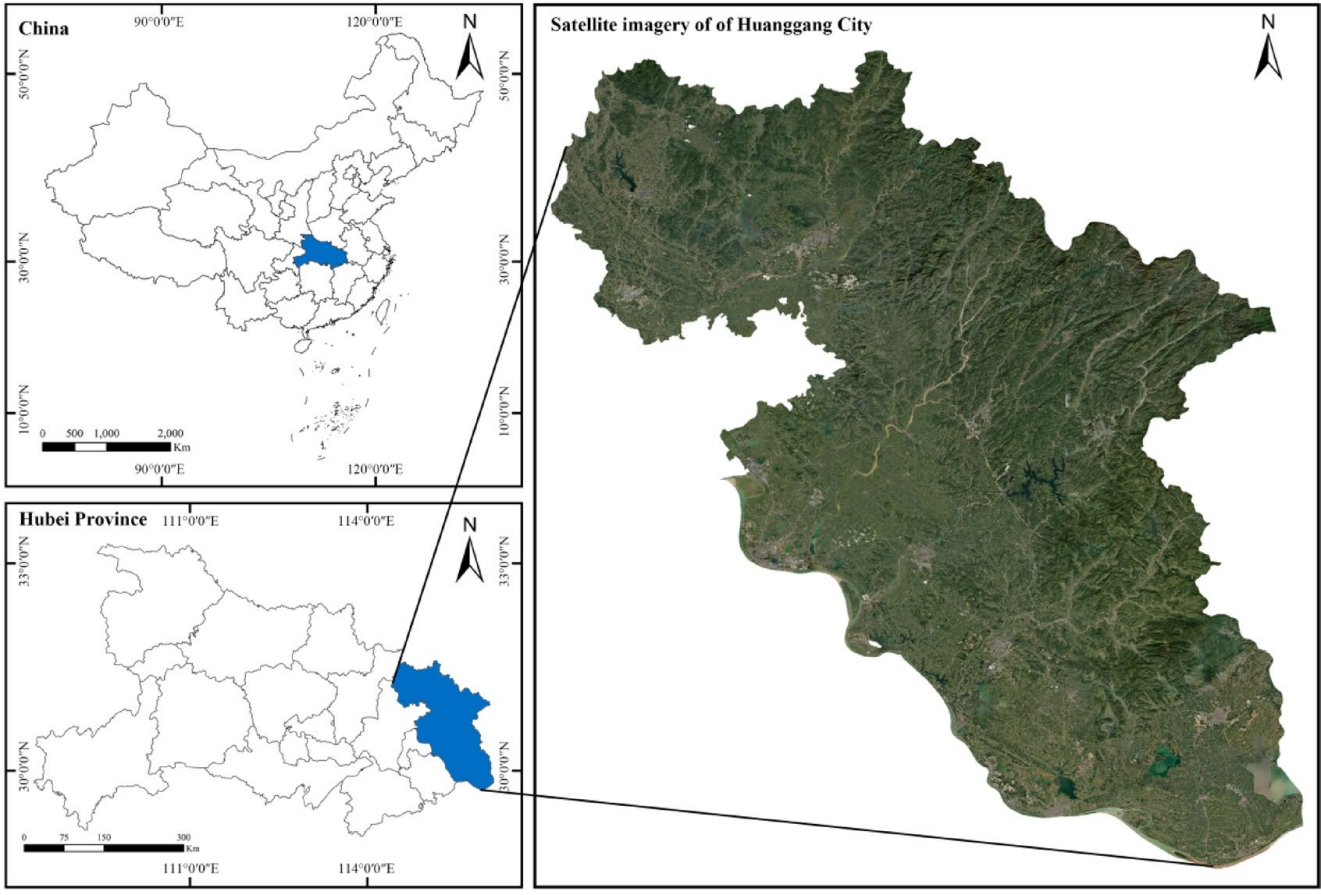
Study area location map. The China Basic Standard Map was obtained from the Standard Map Service website of the Ministry of Natural Resources (https://vgimap.tianditu.gov.cn/), with the review number GS(2024)0650, and the base map has not been modified. This figure was created by the authors using ArcGIS 10.8.1 (Environmental Systems Research Institute, USA. https://www.esri.com/).

Based on the *Catalogue of Celebrity Former Residences among Immovable Cultural Relics in Huanggang City* provided by the Huanggang Culture and Tourism Bureau, a total of 136 celebrity residences were selected as the research objects for evaluation and analysis, as shown in Table 1. Huanggang City is located in the eastern part of Hubei Province, on the southern foothills of the Dabie Mountains and the northern bank of the middle reaches of the Yangtze River. The geographical coordinates extend from 114°24′ to 116°07′ east longitude and from 29°45′ to 31°40′ north latitude, as shown in Fig. 1. The terrain gradually slopes from north to south. The northeastern part borders Henan and Anhui provinces and is characterized by the Dabie mountain range. The central region is dominated by hilly landscapes with alternating elevations, while the southern part is composed of narrow plains and lake areas^38^.

**Table 1:** Distribution of 136 existing cultural heritage protection units of celebrity former residences in Huanggang city.

	Administrative unit	Quantity (Units)	L1	L2	L3	L4	L5
Huanggang city, Hubei province	Xishui county	1	-	-	-	-	1
	Hong’an county	72	2	6	-	60	4
	Huangzhou district	2	-	1	-	1	-
	Tuanfeng county	7	-	3	-	4	-
	Wuxue city	1	-	-	-	1	-
	Luotian county	3	-	1	-	-	2
	Macheng city	33	-	-	-	26	7
	Yingshan county	2	-	-	2	-	-
	Qichun county	3	-	1	-	-	2
	Huangmei county	12	-	2	1	8	1
		136	2	14	3	100	17

L1: National key cultural relics protection unit; L2: Provincial-level cultural relics protection unit; L3: City/prefecture/forest cultural relics protection unit; L4: County/city/district cultural relics protection unit; L5: Not yet designated protection unit.

### Research framework

**Fig. 2:**
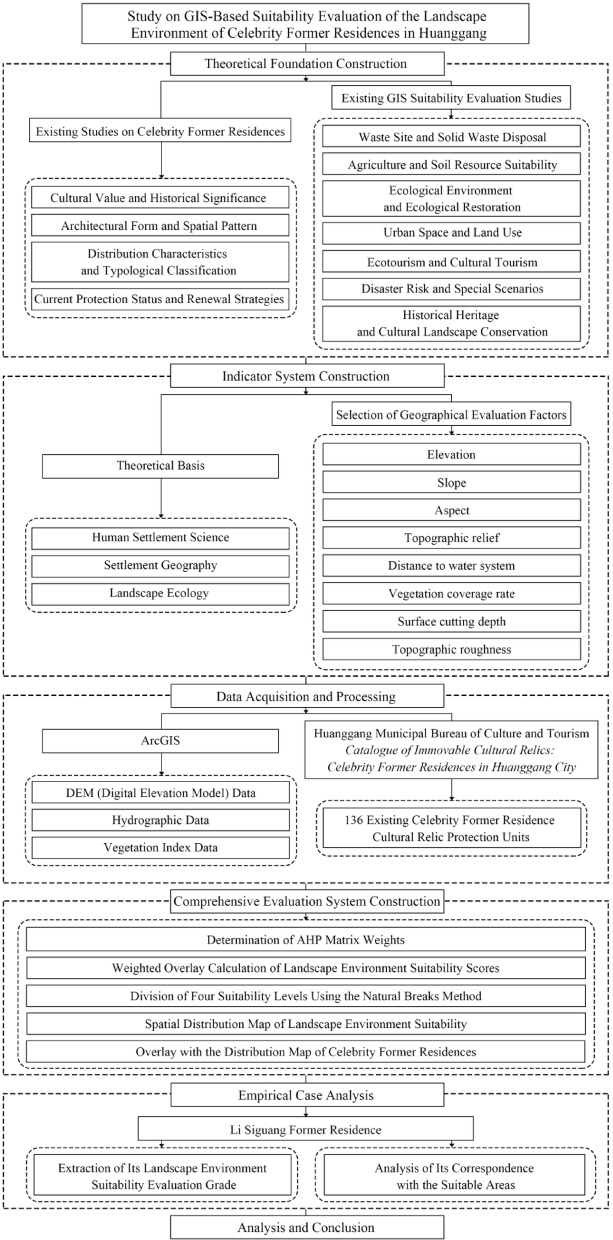
Research framework.

After selecting eight geographical evaluation factors, this study applied the AHP to calculate the weights of each factor and employed ArcGIS for weighted overlay analysis. This approach enabled a suitability evaluation of the landscape environment for the siting of celebrity residences in Huanggang City. The overall research framework is illustrated in Fig. 2.

AHP, first introduced by Thomas L. Saaty in 1980, is one of the most widely used and influential methods for solving multi-criteria decision-making problems. Its core principle is to decompose complex problems into multiple levels and factors, conduct pairwise comparisons among factors, and establish a judgment matrix. By calculating the maximum eigenvalue and eigenvector, the relative importance weights of various factors can be determined, thereby providing a scientific basis for optimal decision-making^39^.

### Selection of geographical evaluation factors

From a geographical perspective, environmental suitability is closely related to settlement siting. Different theoretical frameworks offer scientific guidance for selecting evaluation factors. In this study, theories of human settlement science, settlement geography, and landscape ecology were integrated to identify eight geographical evaluation factors for analysis.

Human settlement science focuses on the relationship between the living environment and human well-being, emphasizing adaptation and harmony between people and their natural surroundings^40^. A suitable residential environment requires favorable topographic conditions, an appropriate climate, and a balanced ecological support system^41^. Accordingly, elevation, slope, and aspect were chosen as key evaluation factors. Elevation reflects differences in temperature, humidity, and climatic comfort, directly influencing living conditions. Slope determines whether land is suitable and safe for construction, as overly steep or excessively flat slopes compromise environmental stability. Aspect affects sunlight exposure and heat distribution, which are crucial for residential comfort.

Settlement geography emphasizes the coupling between spatial settlement patterns and natural environmental conditions^42^. Settlement distribution is not random but reflects an integrated response to terrain, hydrology, and surface resources^43^. For this reason, topographic relief, surface cutting depth, and distance to water systems were selected as additional evaluation factors. Topographic relief reveals the general geomorphological framework of settlements and their spatial relationship with mountain ridges and river orientations. Surface cutting depth reflects landform stability, where excessive cutting hampers settlement formation, while moderate undulation enhances stability and safety. Distance to water system is a core factor, indicating both the dependence of settlements on water resources and their connections to transportation, production, and ecology.

Landscape ecology highlights the interaction between landscape patterns, ecological processes, and environmental stability, particularly the role of ecosystem services in supporting settlement development^44^. Based on this perspective, topographic roughness and vegetation coverage rate were chosen as evaluation factors. Topographic roughness represents the complexity of micro-topography, influencing both ecological stability and landscape diversity. Vegetation coverage rate serves as an indicator of ecosystem quality, where higher coverage reflects stronger soil and water conservation, climate regulation, and greater environmental and landscape benefits.

In summary, this study integrates the requirements of human settlement suitability, the coupling of settlement patterns with natural environmental conditions, and the interaction between ecosystem functions and landscape structures. Eight geographical evaluation factors were selected to represent topography, hydrology, ecological conditions, and geomorphological stability, as shown in Table 2. These include elevation, slope, aspect, topographic relief, surface cutting depth, topographic roughness, vegetation coverage rate, and distance to water system. Together, they constitute a scientific evaluation index system that provides the foundation for a systematic analysis of the suitability of the landscape environment of celebrity residences in Huanggang City.

**Table 2:** Selection of geographical evaluation factors and their data sources.

Category	Geographical Evaluation Factor	Data Source	Coordinate System	Spatial Resolution
Topography	Elevation	DEM (Digital Elevation Model)data (https://www.gscloud.cn/)	WGS_1984_UTM_Zone_50N	30 m
	Slope	DEM (Digital Elevation Model)data (https://www.gscloud.cn/)	WGS_1984_UTM_Zone_50N	30 m
	Aspect	DEM (Digital Elevation Model)data (https://www.gscloud.cn/)	WGS_1984_UTM_Zone_50N	30 m
	Topographic relief	DEM (Digital Elevation Model)data (https://www.gscloud.cn/)	WGS_1984_UTM_Zone_50N	30 m
Hydrological Environment	Distance to water system	Water system data (https://www.tianditu.gov.cn/)	WGS_1984_UTM_Zone_50N	30 m
Ecological Conditions	Vegetation coverage rate	Vegetation index data (https://www.resdc.cn/)	WGS_1984_UTM_Zone_50N	Vector data
Geomorphological stability	Surface incision depth	DEM (Digital Elevation Model)data (https://www.gscloud.cn/)	WGS_1984_UTM_Zone_50N	30 m
	Terrain roughness	DEM (Digital Elevation Model)data (https://www.gscloud.cn/)	WGS_1984_UTM_Zone_50N	30 m

Among the eight evaluation factors selected in this study, topographic factors such as elevation, slope, aspect, terrain relief, surface incision depth, and terrain roughness were all extracted from DEM data. As structural geomorphological indicators, they exhibit minimal short-term variation and strong spatiotemporal stability, so the use of data from different years has limited impact on the evaluation results. For factors with temporal variability, such as vegetation coverage and distance to water bodies, data sources closest in time to the DEM were preferentially selected, and all datasets were processed with a unified projection coordinate system and spatial resolution to ensure spatial comparability. Prior to the comprehensive evaluation, all factors were standardized and subjected to weighted overlay analysis, which methodologically minimizes the influence of temporal discrepancies on the results.

### GIS-based AHP for comprehensive site selection and landscape suitability evaluation

#### Evaluation and analysis of geographical factors for site selection

**Fig. 3:**
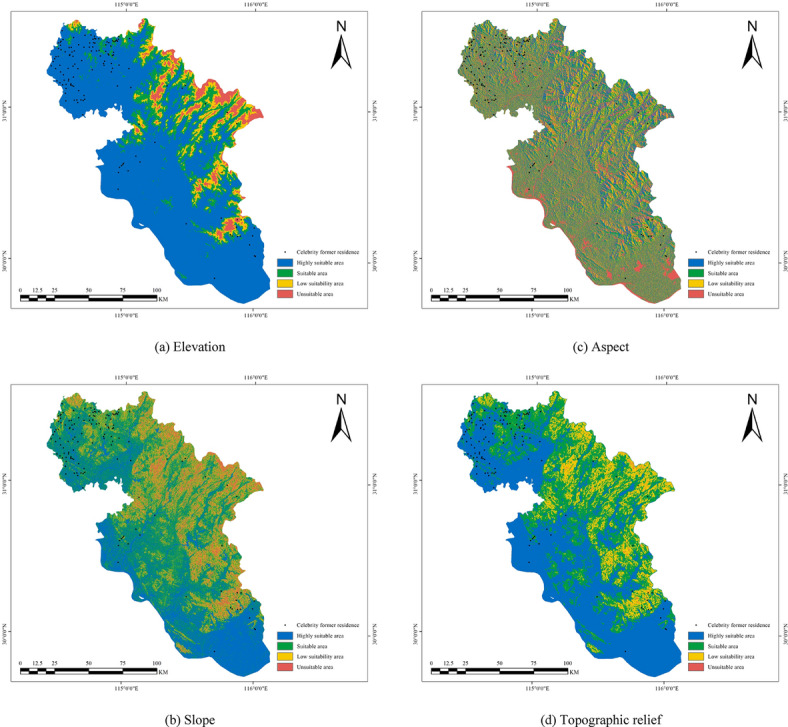

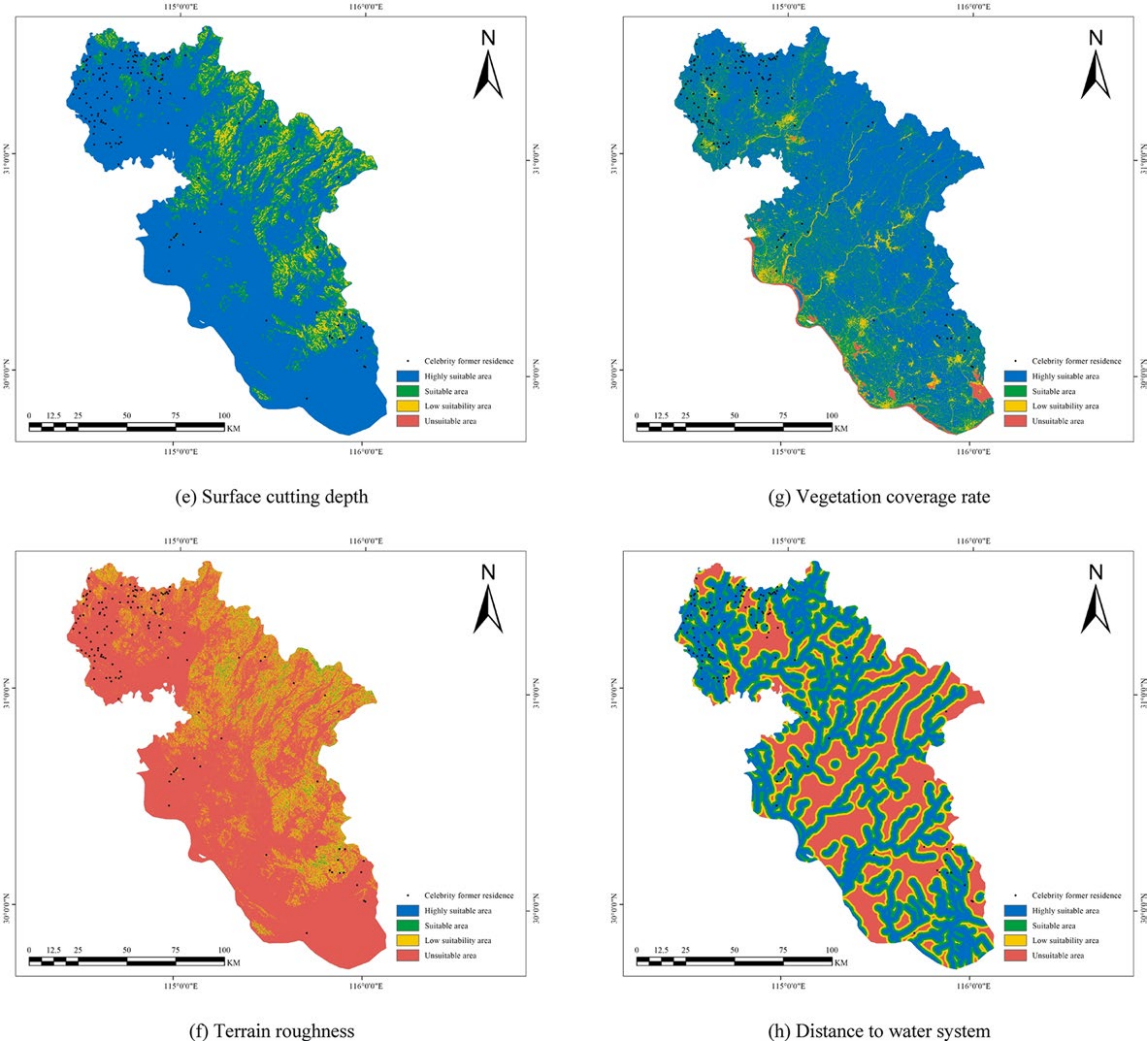
Evaluation maps of landscape environmental suitability for various geographic assessment factors. This figure was created by the authors using ArcGIS 10.8.1 (Environmental Systems Research Institute, USA. https://www.esri.com/).

This study determines the grading standards for each geographical evaluation factor based on threshold ranges commonly adopted in studies of human settlement suitability^19,45^, land suitability analysis^46^, and comprehensive spatial environmental evaluation^26^, and conducts suitability classification through a multi-indicator grading system. On this basis, and in combination with the topographic characteristics of Huanggang City reflected in the *Huanggang Statistical Yearbook (2024)*, the grading intervals of each indicator are regionally adjusted and calibrated to ensure consistency between the theoretical basis and regional applicability of the evaluation system (as shown in Table 3). The spatial distribution of these factors was further analyzed through visualization using ArcGIS. Location data for celebrity former residences were obtained from the Catalogue of Celebrity Former Residences among Immovable Cultural Relics in Huanggang City. Their spatial coordinates were verified and corrected using Amap (https://ditu.amap.com/) and Tianditu (https://www.tianditu.gov.cn/) to ensure the accuracy and reliability of the data. By integrating the distribution of 136 officially protected celebrity former residences within Huanggang City, a series of spatial analysis maps for each geographical evaluation factor were generated, as illustrated in Fig. 3. These maps provided the foundation for assessing the suitability of the landscape environment.

**Table 3:** Geographical evaluation factor grading table.

Geographical evaluation factor	Grading standard	Suitability area level	Score
Elevation	0–200 m	Highly suitable area	4
	200–400 m	Suitable area	3
	400–600 m	Low suitability area	2
	> 600 m	Unsuitable area	1
Slope	0°−5°	Highly suitable area	4
	5°−15°	Suitable area	3
	15°−25°	Low suitability area	2
	> 25°	Unsuitable area	1
Aspect	Flat slope, sunny slope	Highly suitable area	4
	Semi-sunny slope	Suitable area	3
	Semi-shady slope	Low suitability area	2
	Shady slope	Unsuitable area	1
Topographic relief	< 50 m	Highly suitable area	4
	50–150 m	Suitable area	3
	150–300 m	Low suitability area	2
	> 300 m	Unsuitable area	1
Surface cutting depth	< 50 m	Highly suitable area	4
	50–100 m	Suitable area	3
	100–200 m	Low suitability area	2
	> 200 m	Unsuitable area	1
Topographic roughness	> 1.5	Highly suitable area	4
	1.2–1.5	Suitable area	3
	1.05–1.2	Low suitability area	2
	< 1.05	Unsuitable area	1
Vegetation coverage rate	> 80%	Highly suitable area	4
	50%−80%	Suitable area	3
	20%−50%	Low suitability area	2
	< 20%	Unsuitable area	1
Distance to water system	0–1 km	Highly suitable area	4
	1–2 km	Suitable area	3
	2–3 km	Low suitability area	2
	> 3 km	Unsuitable area	1

### Establishment of a landscape suitability evaluation framework for celebrity former residences in Huanggang City

To further explore the site-selection patterns and landscape characteristics of celebrity former residences in Huanggang, a systematic evaluation framework was constructed following the extraction and analysis of the eight geographical evaluation factors. This framework was designed to quantify the influence of geographical conditions on residence site selection, thereby offering both theoretical support and empirical evidence for scientifically evaluating the rationality of site selection and the suitability of the surrounding landscape environment.

AHP was employed to calculate the relative weights of each factor. By constructing pairwise comparison matrices, the method highlights the relative importance of different evaluation factors and facilitates the transformation of subjective judgments into consistent quantitative weights. In this study, five university professors with expertise in GIS, landscape planning, cultural heritage conservation, and environmental assessment were invited to participate in the scoring process. Considering the specific conditions of Huanggang City and incorporating the expert judgments, a pairwise comparison matrix based on the eight geographical evaluation factors was developed, as shown in Table 4.

**Table 4:** AHP judgment matrix for each geographical evaluation factor.

Evaluation factor	F1	F2	F3	F4	F5	F6	F7	F8
F1	1	1/5	1/7	1/5	1/6	1/6	1/3	1/9
F2	5	1	1/4	1/2	1/3	1/3	2	1/6
F3	7	4	1	3	2	2	4	1/3
F4	5	2	1/2	1	1/2	1/2	3	1/5
F5	6	3	1/2	2	1	1/2	3	1/4
F6	6	3	1/2	2	2	1	3	1/4
F7	3	1/2	1/4	1/3	1/3	1/3	1	1/7
F8	9	6	3	5	4	4	7	1

F1: Elevation, F2: Slope, F3: Aspect, F4: Topographic relief, F5: Surface cutting depth, F6: Terrain roughness, F7: Vegetation coverage rate, F8: Distance to water system.

To ensure the validity of the weight vector in explaining the judgment matrix, a consistency test was performed. The calculation formulas for the Consistency Index *(CI)* and Consistency Ratio *(CR)* are as shown in Eqs. (1) and (2), respectively:

1 $$\:\begin{array}{c}\mathrm{C}\mathrm{I}\mathrm{=}\frac{{\lambda}_{\mathrm{max}}\mathrm{-n}}{\mathrm{n-1}}\end{array}$$

2 $$\:\begin{array}{c}\mathrm{C}\mathrm{R}\mathrm{=}\frac{\mathrm{CI}}{\mathrm{RI}}\end{array}$$

Where $\:{\lambda}_{\mathrm{max}}$ is the maximum eigenvalue of the judgment matrix, and *n* is the order of the matrix. Subsequently, *CI* is compared with the Random Consistency Index *(RI)* to obtain *CR*. The calculation results show (as presented in Table 5): $\:{\lambda}_{\mathrm{max}}$ is 8.382, *CI* is 0.055, *RI* is 1.410, and *CR* is 0.039 (< 0.1). Therefore, the judgment matrix passes the consistency test, and the calculated weights are consistent. After normalizing the judgment matrix, the eigenvectors and normalized weights of the eight geographical evaluation factors were obtained, as shown in Table 6.

**Table 5:** Summary of consistency test results.

Maximum eigenvalue	CI	RI	CR	Consistency test result
8.382	0.055	1.410	0.039	Passed

**Table 6:** Standardized weight values of each geographical evaluation factor.

Geographical evaluation factor	Eigenvector	Weight value (%)
Elevation	0.17	2.08
Slope	0.47	5.89
Aspect	1.48	18.48
Topographic relief	0.66	8.28
Surface cutting depth	0.91	11.31
Terrain roughness	1.06	13.23
Vegetation coverage rate	0.33	4.17
Distance to water system	2.93	36.56

### Evaluation system results and analysis

By overlaying the evaluation maps of the eight geographical factors with the distribution map of celebrity former residences in Huanggang City, the results demonstrate a strong correspondence between the residences’ spatial distribution and the suitability levels of the geographical evaluation factors, as summarized in Table 7.

**Table 7:** Suitability level areas for each geographical evaluation factor.

Geographical evaluation factor	Suitability level	Proportion of suitable area (%)
Elevation	4	71.39
	3	15.87
	2	7. 89
	1	4.85
Slope	4	34.63
	3	40.14
	2	17.06
	1	8.17
Aspect	4	24.67
	3	25.21
	2	23.95
	1	26.17
Topographic relief	4	50.40
	3	35.86
	2	13.48
	1	0.26
Surface cutting depth	4	74.86
	3	20.20
	2	4.93
	1	0.01
Terrain roughness	4	0.04
	3	1.95
	2	17.25
	1	80.76
Vegetation coverage rate	4	68.61
	3	23.95
	2	5.62
	1	1.82
Distance to water system	4	33.74
	3	24.84
	2	16.99
	1	24.43

In terms of “elevation”, 71.39% of residences are located in areas below 200 m, classified as highly suitable zones. In terms of slope, 74.77% are distributed within areas of less than 5° (high suitability area) or between 5° and 15° (suitable). Aspect analysis shows a relatively balanced distribution, with 49.88% situated on sunny slopes (high suitability) and semi-sunny slopes (suitable). For relief amplitude, 50.40% are located in areas with less than 50 m of variation, considered highly suitable. With respect to surface dissection, 74.86% of the residences fall within areas of less than 50 m, also classified as highly suitable. Although many residences are found in zones where terrain roughness values are below 1.05, considered unsuitable, a combined 19.24% are distributed across zones with values of 1.05–1.20 (low suitability), 1.20–1.50 (suitable), and above 1.50 (high suitability), which still provides meaningful reference for evaluating landscape suitability. For vegetation coverage, 68.61% are located in areas exceeding 80%, reflecting highly suitable environmental conditions. Regarding proximity to water systems, the distribution is relatively balanced, yet 58.58% are located within 0–1 km (high suitability) and 1–2 km (suitable), exceeding the proportion in areas 2–3 km (low suitability) and beyond 3 km (unsuitable).

**Fig. 4:**
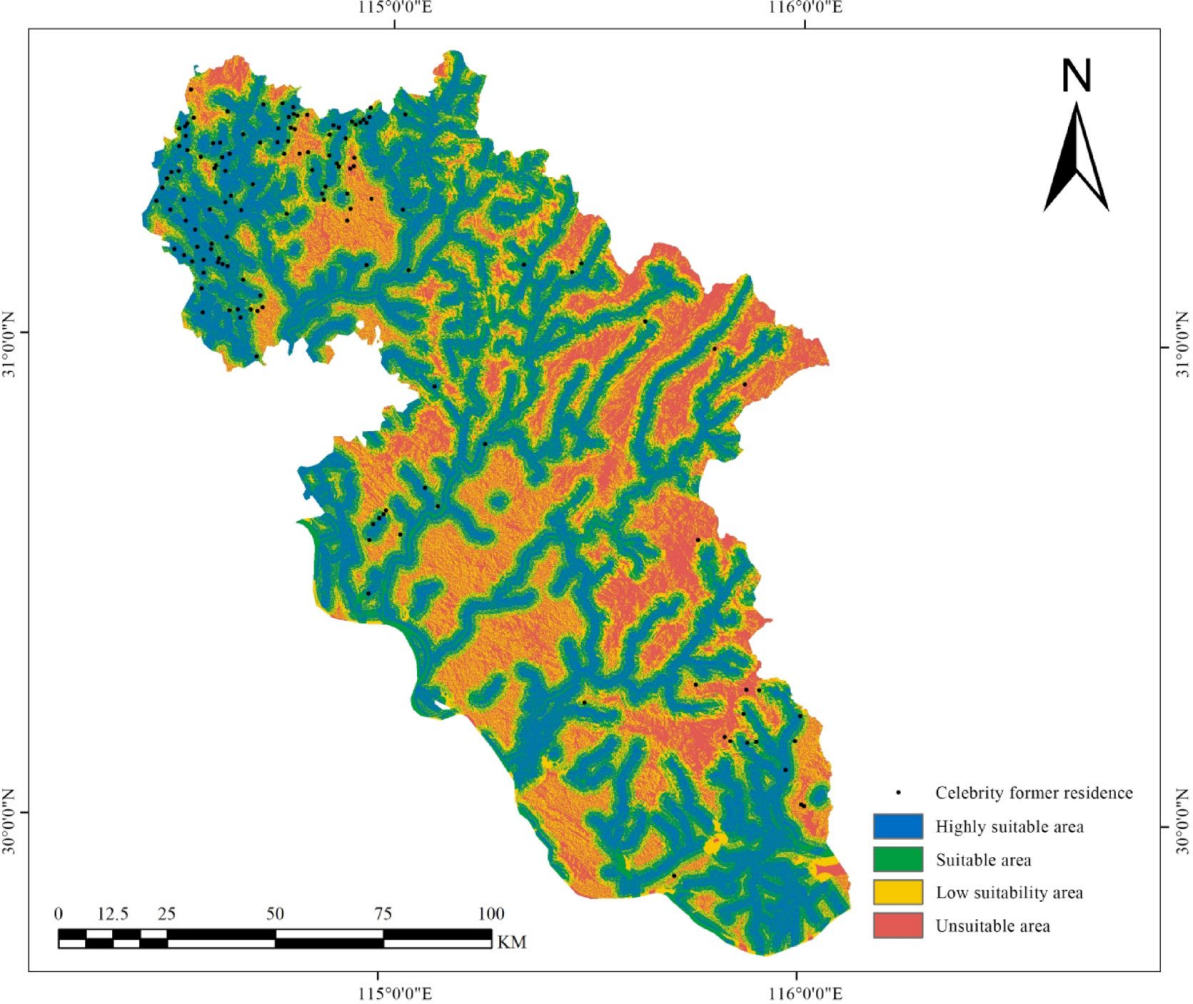
AHP-GIS suitability evaluation map of Huanggang city. This figure was created by the authors using ArcGIS 10.8.1 (Environmental Systems Research Institute, USA. https://www.esri.com/).

Based on the grading standards of each geographical evaluation factor in Table 3, weights were assigned to the indicators, and a weighted overlay analysis was performed in ArcGIS to calculate the comprehensive suitability index of the landscape environment for celebrity former residences in Huanggang City, with a total score ranging from 1.18 to 3.74. The comprehensive index was then classified using the natural breaks (Jenks) method into four levels: highly suitable (3.00–3.74), suitable (2.30–2.99), low suitability (1.70–2.29), and unsuitable (1.18–1.69). Finally, by overlaying the distribution of celebrity former residences in Huanggang City, an AHP-GIS based evaluation map of landscape environmental suitability was generated, as shown in Fig. 4. The results indicate that 66 residences, accounting for 48.53% of the total, are located in highly suitable zones; 49 residences, accounting for 36.03%, fall within suitable zones; 18 residences, accounting for 13.24%, are situated in low suitability zones; and only 3 residences, accounting for 2.20%, are located in unsuitable zones. The weighted overlay model is presented in Eq. (3):

3 $$\:\begin{array}{c}\mathrm{S}\mathrm{(}\mathrm{x}\mathrm{,}\mathrm{y}\mathrm{)}\mathrm{=}{\sum\:}_{\mathrm{i}\mathrm{=1}}^{\mathrm{n}}{\mathrm{w}}_{\mathrm{i}}\cdot{\mathrm{r}}_{\mathrm{i}}\left(\mathrm{x,y}\right)\end{array}$$

Where $\:\mathrm{S(}\mathrm{x,y}\mathrm{)}$ represents the comprehensive suitability score for a given spatial location $\:\mathrm{(}\mathrm{x,y}\mathrm{)}$, $\:\mathrm{i}$ denotes the $\:\mathrm{i}{\:-}\mathrm{th}$ factor, $\:{\mathrm{w}}_{\mathrm{i}}$ refers to the weight of the $\:\mathrm{i}{\:-}\mathrm{th}$ factor, and $\:{\mathrm{r}}_{\mathrm{i}}\left(\mathrm{x,y}\right)$ signifies the standardized score of the $\:\mathrm{i}{\:-}\mathrm{th}$ factor at location $\:\left(\mathrm{x,y}\right)$.

Furthermore, spatial statistical analysis in ArcGIS was employed to calculate the proportional area of each suitability class across the study region. The results indicate that highly suitable areas comprise 24.40% of the total land area in Huanggang City, suitable areas comprise 31.17%, low suitability areas comprise 26.29%, and unsuitable areas comprise 18.14%.

From the perspective of administrative spatial distribution, the celebrity former residences in Huanggang City exhibit clear regional disparities. Hong’an County has the largest number of former residences, with a total of 72, accounting for 52.94% of the total sample, forming a highly concentrated spatial pattern. The suitability evaluation indicates that most of the residences in this area are located within highly suitable and suitable zones, which further confirms the strong environmental adaptability of their traditional site selection in terms of topography, hydrology, and ecological conditions.

The concentration of former residences in Hong’an County is closely related to its distinctive historical development background. As an important revolutionary base area, the region produced a large number of revolutionary figures during modern Chinese history. Consequently, their former residences were often established in hilly and mountainous areas, forming clustered settlement patterns. Such terrain not only provided a certain degree of concealment and defensive advantage, but also corresponded with the traditional principle of siting settlements near mountains and water, resulting in a dense and relatively concentrated distribution of former residences. In contrast, the number of former residences in other counties and districts is relatively small, and they are mostly distributed in a scattered, point-like pattern. Their suitability levels also vary to some extent, reflecting differences in natural geographical conditions and historical development trajectories across regions.

Overall, the analysis reveals that 84.59% of celebrity former residences in Huanggang City are located within highly suitable and suitable zones, demonstrating a strong alignment with the constructed AHP-GIS suitability evaluation system. This highlights the critical role of the eight geographical evaluation factors in residence site selection and underscores the scientific validity and rationality of the proposed framework.

### Case study – taking Li Siguang’s former residence as an example

#### Introduction to the case study

To further enhance the suitability evaluation of the landscape environment of celebrity former residences in Huanggang City, the former residence of Li Siguang was selected as a case study. By analyzing its spatial environmental conditions and site-selection characteristics, and applying ArcGIS-based calculations of suitability scores, the study quantitatively evaluates the residence’s landscape environment to verify both the rationality of its location and the scientific validity of the evaluation framework.

**Fig. 5:**
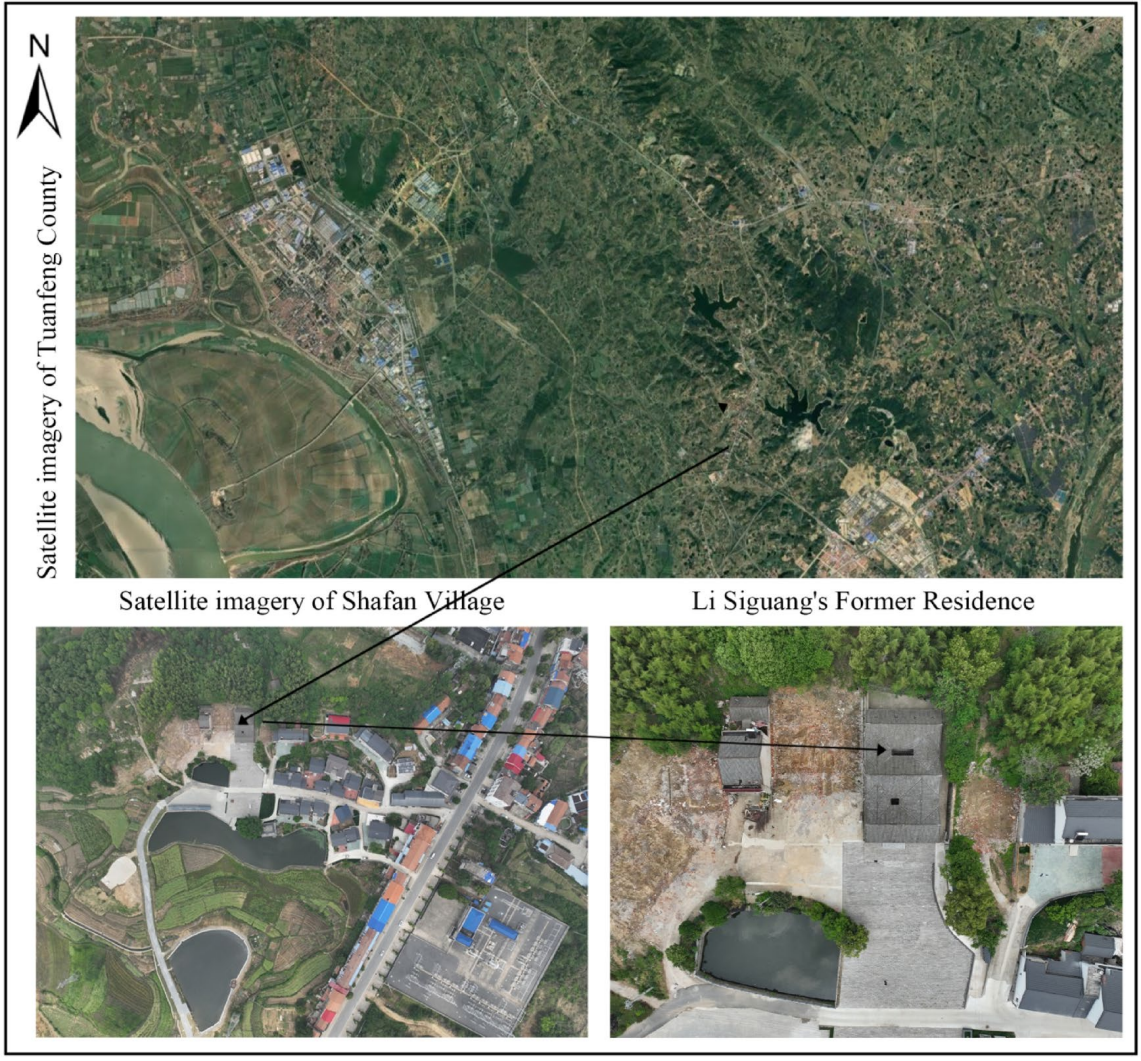
Geographical location analysis of Li Siguang’s former residence. This figure was created by the authors using ArcGIS 10.8.1 (Environmental Systems Research Institute, USA. https://www.esri.com/).

Li Siguang (original name Li Zhongkui), a native of Huanggang, Hubei, was the founder of geomechanics in China and a leading pioneer of modern earth sciences and geological research, widely honored as the “Father of Chinese Geology.” His former residence is located in Shafan Village, west of Huilongshan Town in Tuanfeng County, Huanggang City, at 114.98°E and 30.61°N, on the southern flank of the Dabie Mountains. The site lies east of the Ba River and west of the Sha River and Jushui River, as shown in Fig. 5. According to the Gazetteer of Huangzhou Prefecture, the residence was originally built in 1889, later destroyed and reconstructed, with the current buildings rebuilt in 2006, covering an area of 260 m². On March 27, 2008, the People’s Government of Hubei Province designated it as a provincial-level protected cultural heritage site.

### Evaluation and analysis of Li Siguang’s former residence site selection

When analyzed against the AHP-GIS evaluation system, the former residence demonstrates the following characteristics across the eight geographical evaluation factors, as shown in Table 8:

**Table 8:** Grade standards and suitability levels for each geographical evaluation factor of Li Siguang’s former residence.

Geographical evaluation factors	Grade standards	Suitability grade
Elevation	< 200 m	4
Slope	5°−15°	3
Aspect	Semi-sunny slope	3
Topographic relief	50–150 m	3
Surface cutting depth	< 50 m	4
Terrain roughness	1.05–1.2	2
Vegetation coverage rate	50%−80%	3
Distance to water system	0–1 km	4

1. Elevation: Situated in the transitional zone between the Dabie Mountain foothills and the Yangtze River Plain, with an altitude of approximately 50 m, reflecting a relatively low-lying and habitable terrain.
2. Topographic relief: Minimal variation, with an overall flat landscape conducive to settlement and agricultural activities.
3. Slope: Gentle slopes, favoring structural stability and construction feasibility.
4. Aspect: Located on a semi-sunny slope, ensuring favorable sunlight exposure and ventilation.
5. Topographic relief: Low roughness values, indicating limited micro-topographical fluctuation and overall surface stability.
6. Surface cutting depth: Within a low-value zone, reflecting a stable and undisturbed landscape setting.
7. Vegetation coverage rate: Approximately 68%, offering a balanced ecological environment that regulates the microclimate and enhances the residential landscape.
8. Distance to water system: The site is located at a moderate distance from nearby rivers, ensuring sufficient access to domestic and agricultural water while avoiding excessive flood risks.

**Fig. 6:**
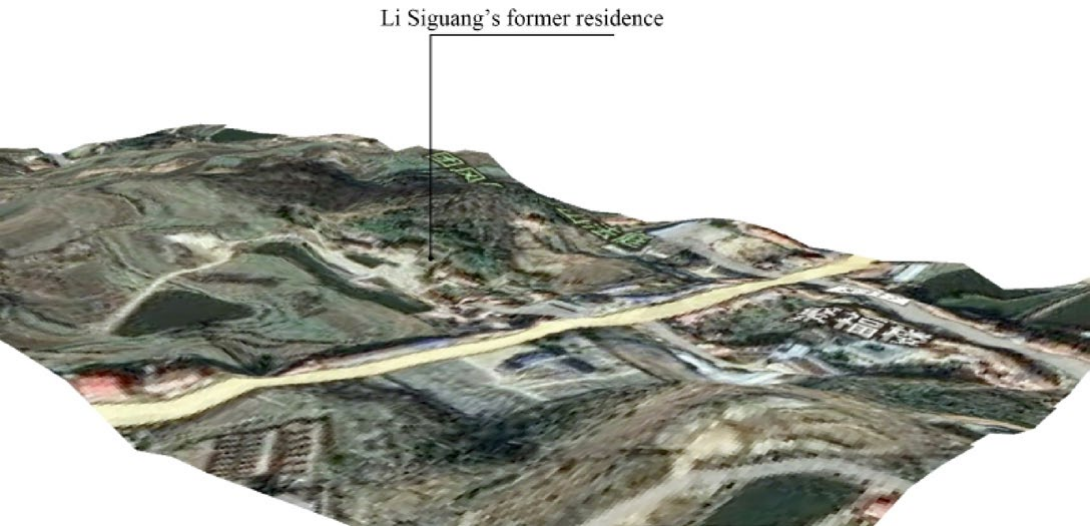
Topographic map of Li Siguang’s former residence. This figure was created by the authors using ArcGIS 10.8.1 (Environmental Systems Research Institute, USA. https://www.esri.com/).

These conditions collectively highlight the residence’s high suitability in terms of the landscape environment, further validating the scientific robustness of the AHP-GIS suitability evaluation framework, as illustrated in Fig. 6.

### Evaluation results and analysis of Li Siguang’s former residence

Based on calculations using the AHP-GIS landscape suitability evaluation framework, the former residence of Li Siguang obtained a comprehensive site-selection score of 3.37, classifying it within the “high suitability zone.” This result demonstrates a strong correspondence between the site’s spatial environmental pattern and the grading standards of the geographical evaluation factors.

From the above combination of evaluation factors, the geographical advantages of Li Siguang’s former residence are mainly reflected in two dimensions: terrain stability and water accessibility. Low elevation, gentle slopes, and relatively small terrain dissection depth together create comparatively stable geological conditions. A moderately close distance to water sources not only satisfies daily water needs but also reduces flood risk. Meanwhile, medium-to-high vegetation coverage and moderate terrain roughness contribute to a favorable microclimate and ecological foundation. The integrated configuration of these environmental factors reflects the comprehensive consideration of safety, resource accessibility, and ecological conditions in traditional settlement site selection, and further verifies the relationship between the distribution of famous figures’ former residences and natural geographical factors. The factor-combination pattern demonstrated in this case also provides a reference for identifying common spatial characteristics of former residences in other highly suitable areas of Huanggang.

## Discussion

This study constructs an evaluation framework based on the AHP and GIS to provide a more scientific method for assessing the landscape environmental suitability of celebrity former residences in Huanggang City. By integrating multiple geographic factors—including elevation, slope, aspect, topographic relief, surface cutting depth, topographic roughness, vegetation coverage rate, and distance to water systems—the framework effectively reveals the spatial distribution patterns of these residences and their relationship with natural environmental attributes. The results show that among 136 former residences, 66 are located in highly suitable zones, underscoring the critical role of favorable geomorphological and hydrological conditions in traditional settlement site selection. The case study of Li Siguang’s former residence further validates the stability of the proposed evaluation framework, as its actual location aligns closely with the highly suitable zones identified by the model.

The evaluation framework constructed in this study classifies landscape suitability into four levels: highly suitable areas (3.00–3.74), primarily distributed near foothills and rivers, which should prioritize both cultural heritage protection and appropriate landscape utilization; suitable areas (2.30–2.99), where targeted landscape optimization can be implemented to enhance connectivity and accessibility while maintaining the integrity of historical environments; low suitability areas (1.70–2.29), which require environmental restoration or the establishment of protective buffers to mitigate adverse impacts; and unsuitable areas (1.18–1.69), mainly designated for ecological conservation or indirect heritage management strategies. By overlaying the distribution of former residences with the suitability map, the framework provides a quantifiable and actionable reference for cultural heritage protection and planning.

Compared with previous studies on human settlement, agricultural land, or other site suitability assessments, this study presents several theoretical innovations: first, it introduces a cultural heritage dimension into traditional GIS-based natural environment evaluation, enabling a quantitative analysis of the spatial distribution of historical sites and conservation planning; second, it applies regionalized thresholds, adapting the standard AHP-GIS methodology to the topographic characteristics of Huanggang City, thereby improving the local applicability of the model; third, by overlaying the distribution of former residences with the suitability map, it provides data-driven decision support for heritage management, integrating geographic analysis with cultural landscape conservation practice.

### Suitability-based differentiated management strategies

Based on the suitability evaluation results and spatial distribution of celebrity former residences in this study, differentiated management strategies should be implemented according to the suitability levels of different areas:

1. **Highly Suitable Areas**: Given the advantages of natural environment and historical context, these areas should be given priority protection. Large-scale development should be strictly controlled to maintain the existing “mountain–water–village–residence” spatial pattern. Surrounding natural features (such as ancient trees and water systems) and traditional building textures should be restored, and buffer zones established to prevent urban encroachment.
2. **Suitable Areas**: These areas have a certain natural foundation, but landscape continuity and historical atmosphere have been partially disrupted. Organic updates and restorations can be applied to modern buildings that conflict with traditional features. Entrance landscapes, pavement, and green spaces should be improved to enhance the recognizability and accessibility of the residences.
3. **Low Suitability Areas**: Natural terrain features are weak in these areas, which are often located in modern town centers or along main transportation routes. Existing residences should be protected primarily at the building level, focusing on interior exhibitions rather than reconstructing the surrounding historical environment. Alternatively, they can serve as urban cultural nodes through localized development that integrates modern landscape design.
4. **Unsuitable Areas**: These areas are not suitable for direct development or restoration. Emphasis should be placed on ecological protection and landscape buffering. Existing residences can be digitally archived and designated as cultural landmarks to preserve their historical context.

### Regional coordinated protection strategies

Regarding the distribution of residences and cultural resources across administrative regions, coordinated regional protection strategies should be implemented:

1. **Areas with high residence density (e.g.**,** Hong’an County)**: Cross-administrative “Celebrity Residence Cultural Ecological Corridors” should be established. Scattered residences can be connected via greenways and scenic routes to form linked clusters, shifting from individual site protection to cluster-based revitalization.
2. **Areas with sparse residence distribution but rich cultural heritage (e.g.**,** Huangzhou District)**: A “point activation and heritage continuation” strategy should be adopted. Existing residences should be developed as historical cultural landmarks, carefully integrating them into urban planning to avoid the creation of cultural islands during urban renewal.

## Limitations and future work

However, this study still has certain limitations. The selection of evaluation factors was primarily based on natural elements, with insufficient consideration of socio-cultural attributes (such as family traditions, political and cultural backgrounds, historical significance, and symbolic value) and accessibility, as well as a lack of detailed justification for the choice of factors and their local applicability. The framework primarily relies on natural geographic and topographic conditions because the natural environment imposes fundamental constraints on the site selection of historical celebrity residences, with terrain, hydrology, and aspe ct directly influencing settlement safety, agricultural productivity, and landscape livability. In addition, natural factor data are relatively easy to obtain and quantify, and most historical residences in Huanggang City are located in areas with stable natural conditions, making natural factors the core basis for constructing the evaluation system.

Furthermore, the framework was empirically tested only in Huanggang City, and its applicability in other regions remains to be verified. Future research could expand the evaluation system by incorporating non-natural factors such as socio-cultural attributes, historical value, and accessibility, integrating them with natural conditions for a multidimensional analysis. Additionally, data-driven dynamic weighting methods could be explored, and high-resolution remote sensing, 3D GIS, and virtual reality technologies could be employed for more refined spatial analysis. Comparative studies across regions would also help identify general patterns and local characteristics in heritage site selection, thereby enhancing the scientific rigor and local applicability of the evaluation framework.

## Conclusions

From a geographical perspective, this study employed GIS to evaluate and analyze the landscape site selection of celebrity former residences in Huanggang City. By integrating the AHP, an AHP-GIS suitability evaluation framework was constructed based on eight geographical factors: elevation, slope, aspect, relief amplitude, surface dissection depth, terrain roughness, vegetation coverage, and distance to water systems. Through weight calculation, standardized scoring, and weighted overlay analysis, a comprehensive suitability evaluation map of the study area was generated. The key findings are as follows:

1. **Strong alignment with natural geographical patterns.** The spatial distribution of celebrity former residences demonstrates a clear preference for areas characterized by proximity to mountains and rivers, gentle topography, and sun-facing, sheltered orientations. These findings are consistent with the theoretical foundations of human settlement studies, settlement geography, and landscape ecology.
2. **Validation of the AHP-GIS evaluation framework.** The constructed system effectively reveals the spatial distribution patterns of celebrity former residences. A case study of Li Siguang’s former residence yielded a suitability score of 3.37, placing it in the “high suitability zone,” which highlights favorable topographical and hydrological conditions and confirms the reliability of the evaluation framework.
3. **Quantitative basis for heritage conservation.** The suitability evaluation results show that the “high suitability” and “suitable” zones together account for 84.56% of all identified sites. This provides a scientific reference for guiding future cultural heritage conservation, contextual reconstruction, and adaptive reuse strategies in Huanggang City and beyond.

## Data Availability

The methodological appendices, including Analytic Hierarchy Process (AHP)-based weight determination and the GIS-based suitability evaluation workflow, have been deposited in the Zenodo repository ([https://doi.org/10.5281/zenodo.19665851]). All these datasets, which are publicly available, were compiled and processed by the authors for use in the present study. Elevation, slope, aspect, topographic relief, surface incision depth, and terrain roughness data are publicly available from the [Geospatial Data Cloud Platform][http://www.gscloud.cn]. Distance to the water system data are publicly available from the [Water System Data, Tianditu][https://www.tianditu.gov.cn]. Vegetation coverage rate data are publicly available from the [Resources and Environmental Science Data Center][https://www.resdc.cn]. All figures in this study were created by the authors using the relevant data.
